# GENTLE MOVES: AN INNOVATIVE IN-HOME PHYSICAL ACTIVITY PROGRAM INCLUSIVE OF OLDER ADULTS WITH ADRD

**DOI:** 10.1093/geroni/igae098.4199

**Published:** 2024-12-31

**Authors:** Elise Hu, Lijuan Yin, Woojin Song, Jordan Skowronski, Jane Stocks, Hanaan Bing-Canar, Maria Caceres, Naoko Muramatsu

**Affiliations:** University of Illinois at Chicago, Chicago, Illinois, United States; University of Illinois Chicago, Chicago, Illinois, United States; University of Illinois Chicago, Chicago, Illinois, United States; University of Illinois Chicago, Chicago, Illinois, United States; Northwestern University Feinberg School of Medicine, Chicago, Illinois, United States; Icahn School of Medicine at Mount Sinai, New York, New York, United States; University of Illinois Chicago, Chicago, Illinois, United States; University of Illinois Chicago, Chicago, Illinois, United States

## Abstract

While regular physical activity (PA) is vital for older adults’ health, older adults with Alzheimer’s disease and related dementias (ADRD) often find regular PA unattainable due to prolonged inactivity, functional limitations, and/or lack of motivation. Pro-Home(TM) Gentle Moves for Healthy Aging (GM) is an in-home PA program inclusive of inactive/frail older adults with ADRD, adapted from an evidence-based PA program. GM is innovative because (1) GM applies behavioral learning theory to guide learning, managing, and reinforcing regular PA; (2) GM features PA goalsetting to enhance motivation and builds PA into participants’ daily routine; (3) the in-home PA moves are appropriate for inactive/frail older adults with ADRD; and (4) participants receive a personalized tablet to remind and support daily completion of GM. We remotely pilot-tested GM with 8 participants and their caregivers. Participants had a mean age of 79.3 (SD=9.67), 5 were female, and caregivers were participants’ children (n=4), spouses (n=3), or grandchildren (n=1). Despite varying degrees of ADRD, all dyads actively engaged with our interventionist to set PA goals, learn GM, and incorporate GM into their daily routines. Additionally, all dyads completed the 3-month program (100% retention). Participants felt safe doing GM at home and found GM’s intensity appropriate for their abilities. While most participants with mild ADRD independently completed the 3-month intervention with daily tablet use after initial instruction, participants with moderate ADRD required greater caregiver support using the tablet and reminding and motivating participants daily. Future directions include implementing GM into healthcare settings and investigating further motivational strategies.

